# Genetic mapping of complex traits by minimizing integrated square errors

**DOI:** 10.1186/1471-2156-13-20

**Published:** 2012-03-23

**Authors:** Song Wu, Guifang Fu, Yunmei Chen, Zhong Wang, Rongling Wu

**Affiliations:** 1 Department of Applied Mathematics and Statistics, the State University of New York at Stony Brook, Stony Brook, NY 11790, USA; 2 Center for Computational Biology, Beijing Forestry University, Beijing 100083, China; 3 Center for Statistical Genetics, Pennsylvania State University, Hershey, PA 17033, USA; 4 Department of Mathematics, University of Florida, Gainesville, FL 32611, USA

## Abstract

**Background:**

Genetic mapping has been used as a tool to study the genetic architecture of complex traits by localizing their underlying quantitative trait loci (QTLs). Statistical methods for genetic mapping rely on a key assumption, that is, traits obey a parametric distribution. However, in practice real data may not perfectly follow the specified distribution.

**Results:**

Here, we derive a robust statistical approach for QTL mapping that accommodates a certain degree of misspecification of the true model by incorporating integrated square errors into the genetic mapping framework. A hypothesis testing is formulated by defining a new test statistics - energy difference.

**Conclusions:**

Simulation studies were performed to investigate the statistical properties of this approach and compare these properties with those from traditional maximum likelihood and non-parametric QTL mapping approaches. Lastly, analyses of real examples were conducted to demonstrate the usefulness and utilization of the new approach in a practical genetic setting.

## Background

Genetic mapping of quantitative trait loci, or QTLs, plays prominent roles in understanding the genetic basis of many phenotypic variations [1-4]. Depending on the biological nature of the organism and trait studied, several types of mapping populations generated from different experimental crosses can be constructed to map the QTL of interest. Among those, backcross and F2 intercross are probably two of the most widely used techniques and have been applied in many areas, such as maize and mice studies [5-7]. These experimental crosses separate individual gene components, including QTLs, in a controlled manner, which serves as a foundation for QTL mapping. The basic question is how to efficiently and effectively associate a quantitative trait with its corresponding QTLs and subsequently determine their locations and genetic effects through QTL-linked genetic markers. The past two decades have seen tremendous statistical methodological development in this area [8-16]. Usually, one significant assumption required to derive these statistical methods is that the phenotypic values of a trait can be modelled by a known parametric distribution, such as a normal distribution. By estimating the parameters that define the phenotypic distribution of each genotype at a putative QTL and testing their differences among different QTL genotypes within a mixture model framework, the existence of a QTL and its genetic effects can be inferred on the genome. Statistical approaches for parameter estimation with the mixture model are typically derived within the maximum likelihood (ML) context because of many good properties of a ML estimator, such as asymptotical unbiasedness and asymptotical efficiency. Recently, a surge of interest has also been exploded in solving the mixture models by Bayesian approaches [17-19].

**Figure 1:**
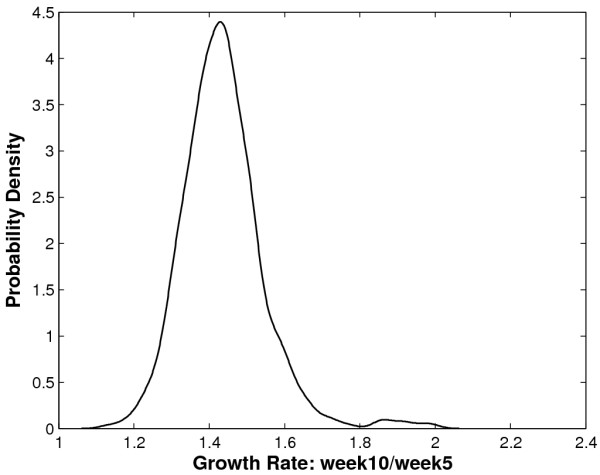
**Mice data density plot**. The empirical density for the growth rate of body mass from ages 5 weeks to 10 weeks in an F_2 _mapping population of 500 mice.

Parametric modelling has the advantage of easy interpretation of results. However, in practice it is often hard or unrealistic to guarantee the assumed model for analysis truly reflects the phenotypic distribution of a trait. For example, significant measurement errors or outliers occurring as a usual case in data collection may lead the observed trait distribution to deviate from the underlying distribution of data. Figure 1 illustrates the empirical density for the growth rate of body mass from ages 5 weeks to 10 weeks in an F_2 _mapping population of 500 mice derived from two inbred lines [20]. It is obvious that the density function is not a perfect normal distribution, as it contains a small bump on the upper tail of the density function. Since the main body of the density curve resembles a normal distribution, the true distribution of observed body mass data (Figure 1) can be viewed as a distorted normal. In these cases, if the traditional methods, such as maximum likelihood, were applied, significant bias would be introduced by the potential outliers. Therefore, there is a pressing need to develop a more robust statistical approach for mapping those complex traits that display such a distribution. Non-parametric rank-based method has been introduced for mapping traits with outliers [11]; however, as it is nonparametric, the interpretability of the mapping results, especially on the genetic effects such as percentage of variance explained by the significant QTL, is usually poor.

In this article, we derive a new mapping approach that is not only robust for genetic mapping of complex traits with the distorted normal distributions, as shown in Figure 1, but also maintains the easy interpretability of a parametric model by minimizing the integrated square errors. The integrated square error has been typically used as the goodness-of-fit criterion in nonparametric density estimation [21]. Some previous studies have also shown that this criterion can be applied in parametric settings and the parameter estimator from this method, or the L_2 _estimator (L_2_E), is robust to the model specification [22-25]. In this sense, this method allows moderate deviation of a proposed density function from the true underlying density. Here, we incorporate the principal of the integrated square errors into genetic mapping framework in a parametric way, and call it the L_2_E mapping method. The main advantage of this new mapping method is that it automatically manipulates data points that are apparently outliers by giving them less weight in parameter estimation, and therefore yields more accurate estimation of QTL locations and effects. In the case where the data cleaning is not possible or very hard to do so, L_2_E method would be a very beneficial choice.

## Methods

### Mapping population

Suppose there is an F_2 _population of *N *progenies, initiated with two different inbred lines, in which there are three groups of genotypes at each gene. A genetic linkage map is constructed for this population, aimed to identify trait-controlling QTLs on the genome. Let y_i _denote a phenotypic trait of interest for F_2 _progeny *i*. It is assumed that a QTL with allele *Q *and *q *exists to affect this trait. The QTL is bracketed by two flanking markers *M_1 _*(with alleles M_1 _and m_1_) and *M_2 _*(with alleles M_2 _and m_2_). Let r_1_, r_2 _and r be the recombination fractions between *M_1 _*and the QTL, between the QTL and *M_2_*, and between the two markers, respectively. Although QTL genotypes are not known, the probability with which a progeny *i *carries a specific QTL genotype can be inferred from the marker genotypes of this progeny. The conditional probability of QTL genotype *j *(*j *= 2 for *QQ*, 1 for *Qq*, and 0 for *qq*), conditional upon one of the nine genotypes of the flanking markers for progeny *i *in the F_2 _population, can be derived and expressed as a function of the recombination fractions (*r*_1_, *r*_2 _and *r*) [26].

Suppose each QTL genotype *j *has a genotypic mean g_j_. The comparisons of these means among three different genotypes can determine whether and how this putative QTL affects the trait. The trait phenotype of progeny *i *due to the QTL can be expressed by the following linear statistical model:

(1) $$y_{i}=\overset{2}{\underset{j=0}{\sum }}\xi_{ij}g_{j}+e_{i}$$

Where *ξ_ij _*is an indicator variable for individual *i *that is defined as 1 for QTL genotype *j *considered and 0 otherwise, and *e_i _*~ *f *(*e*) is the residual effect of progeny *i*, including the aggregate effect of polygenes and error effect.

We assume that *f *(*e*) is the true density of e_i_, which is unknown but has zero mean. Then, the density of y_i _would be a mixture of *f *with mean g_j_. Within the maximum likelihood context, the EM algorithm can be employed to estimate the genetic parameters and test the existence of the QTL [26].

### L_2_E approach

Our proposed L_2_E method is to minimize a data-based estimation of the L_2 _divergence between the assumed model density *φ *and the true objective density (*f*) underlying the data. An energy function (*E) *can be defined to measure the divergence between *φ *and *f*:

$$E=\int |\;\varphi -f{|}^{2}du=\int \varphi^{2}du-2\int \varphi fdu+\int f^{2}du$$

where *u *is a random variable with density of *f*. Since the goal is to minimize E, ∫ *f*^2^*du *can be dropped off because it is a constant of unknown parameters. Hence, the energy function to be minimized can be redefined as

$$E=\int \varphi^{2}du-2\int \varphi fdu=\int \varphi^{2}du-2E\left(\varphi \right)$$

Although it is impossible to give the explicit form of *E*(*φ*), by applying the law of large numbers (LLN), it can be approximated by the observed data and then a new energy function can be formulated as

(2) $$E≅\int \varphi^{2}du-\frac{2}{N}\overset{N}{\underset{i=1}{\sum }}\varphi \left(u_{i}\right)$$

Since the LLN has been employed in the formula derivation, the L_2_E method is not suitable for dataset with a small sample size. Let Θ denote all the parameters in *E*, then the L_2_E of Θ $\text{Θ}\left(\hat{\text{Θ}}\right)$ is

$$\hat{\text{Θ}}=\arg\;\underset{\text{Θ}}{\min}\left(E\right)≅\arg\;\underset{\text{Θ}}{\min}\left(\int \varphi^{2}du-\frac{2}{N}\overset{N}{\underset{i=1}{\sum }}\varphi \left(u_{i}\right)\right)$$

The asymptotic properties of the parameter estimators by L_2_E can be shown by the following proposition.

Proposition 1

*Consider a single trait y. Let φ*(*y *| Θ) *be the parametric model used in (2). Under mild conditions, the L_2_E parameters are consistent and asymptotically normal, i.e.*,

$$\sqrt{n}\left(\hat{\text{Θ}}-{\text{Θ}}_{0}\right)\to N\left(0,A^{-1}\left(B_{2}-B_{1}B_{1}^{T}\right)A^{-1}\right),$$

where

$$A=\int \frac{\partial \varphi }{\partial {\text{Θ}}_{0}}{\left(\frac{\partial \varphi }{\partial {\text{Θ}}_{0}}\right)}^{T}du,B_{2}=\int \frac{\partial \varphi }{\partial {\text{Θ}}_{0}}{\left(\frac{\partial \varphi }{\partial {\text{Θ}}_{0}}\right)}^{T}\varphi du\;\text{and}B_{1}=\int \frac{\partial \varphi }{\partial {\text{Θ}}_{0}}\varphi du$$

Proof

The estimation functions for (2) are

$$\Psi_{n}=\frac{\partial E}{\partial \text{Θ}}=\overset{n}{\underset{i=1}{\sum }}\frac{\partial \varphi \left(u_{i}\right)}{\partial \text{Θ}}-\int \frac{\partial \varphi \left(u\right)}{\partial \text{Θ}}\varphi du=\frac{1}{n}\overset{n}{\underset{i=1}{\sum }}\left(\frac{\partial \varphi \left(u_{i}\right)}{\partial \text{Θ}}-\int \frac{\partial \varphi \left(u\right)}{\partial \text{Θ}}\varphi du\right),\;$$

Define $\psi =\frac{\partial \varphi \left(u\right)}{\partial \text{Θ}}-\int \frac{\partial \varphi \left(u\right)}{\partial \text{Θ}}\varphi du$. Then, from the theory for standard M-estimators, we have

$$\sqrt{n}\left(\hat{\text{Θ}}-{\text{Θ}}_{0}\right)\to N\left(0,A_{\left({\text{Θ}}_{0}\right)}^{-1}B_{\left({\text{Θ}}_{0}\right)}A_{\left({\text{Θ}}_{0}\right)}^{-1}\right),$$

where

$$A\left({\text{Θ}}_{0}\right)=E{\left[-\frac{\partial \psi }{\partial {\text{Θ}}^{T}}\right]}_{\text{Θ}={\text{Θ}}_{0}}=E{\left[\int \frac{\partial^{2}\varphi }{\partial \text{Θ}\partial {\text{Θ}}^{T}}\;du+\int \frac{\partial \varphi }{\partial \text{Θ}}\frac{\partial \varphi }{\partial {\text{Θ}}^{T}}\;du-\int \frac{\partial^{2}\varphi }{\partial \text{Θ}\partial {\text{Θ}}^{T}}\;du\right]}_{\text{Θ}={\text{Θ}}_{0}}=\int \frac{\partial \varphi }{\partial {\text{Θ}}_{0}^{T}}\frac{\partial \varphi }{\partial {\text{Θ}}_{0}}du$$

and

$$B\left({\text{Θ}}_{0}\right)=E{\left[\psi \psi^{T}\right]}_{\text{Θ}={\text{Θ}}_{0}}=\int \frac{\partial \varphi }{\partial {\text{Θ}}_{0}}{\left(\frac{\partial \varphi }{\partial {\text{Θ}}_{0}^{T}}\right)}^{T}\varphi du-\int \frac{\partial \varphi }{\partial {\text{Θ}}_{0}}\varphi du\int \frac{\partial \varphi }{\partial {\text{Θ}}_{0}^{T}}\varphi du.$$

Then, the results follow.

In the setting of genetic mapping, where the density of a mixture of normal applies (model 1), two approaches can be used to implement the principle of minimum integrated squared errors. The most straightforward implementation is to directly model the true density of the error term (e_i_), and the second one is based on modelling the true density of the observed phenotype data (y_i_). The obvious difference between these two methods is that density for e_i _is *f *with mean zero, but the density for y_i _is a mixture of *f *with mean g_j_. A more subtle difference is that the error term based L_2_E method (eL_2_E) involves one additional approximation step in genetic positions between markers. Although simulation studies shown in later sections demonstrate that eL_2_E is inferior to the phenotype data based L_2_E method (pL_2_E), we would still like to present the eL_2_E procedure, because its formulation at marker positions can help derive the pL_2_E method, as will be seen below. Both eL_2_E and pL_2_E employ the energy function *E *defined in Equation (2), with *u *being the error term *e *or the observed data *y*, correspondingly.

### Error term based L_2_E method (eL_2_E)

In model (1), the randomness is derived from the underlying error term. Thus, it is natural to directly model the density of the error term *f(e)*. In a continuous case, a normal density function *φ*(*e*|0,*σ*^2^) is used to approximate the true error density *f(e)*. Thus, using (2), the energy function for error (*E_e_*) becomes

$$E_{e}=\int \varphi^{2}de-\frac{2}{N}\overset{N}{\underset{i=1}{\sum }}\varphi \left(e_{i}\right)$$

where

(3) $$e_{i}=y_{i}-\overset{2}{\underset{j=0}{\sum }}\xi_{ij}g_{j}$$

Notice that

$$\int \varphi^{2}de=\int {\left[\frac{1}{\sqrt{2\pi \sigma^{2}}}e^{-\frac{e^{2}}{2\sigma^{2}}}\right]}^{2}de=\frac{1}{2\sqrt{\pi \sigma^{2}}}$$

Then, the estimators of the unknown parameter set in (Θ = (*g*_0_, *g*_1_, *g*_2_, *σ*^2^)) can be represented as

$$\hat{\text{Θ}}=\arg\;\underset{\text{Θ}}{\min}\left(E_{e}\right)≅\arg\;\underset{\text{Θ}}{\min}\left(\frac{1}{2\sqrt{\pi \sigma^{2}}}-\frac{2}{N}\overset{N}{\underset{i=1}{\sum }}\phi \left(e_{i}\right)\right)$$

where *φ*(*e_i_*) can be approximated by its expectation *E*[*φ*(*e_i_*)]. Based on the error (3), we have

$$E\left[\varphi \left(e_{i}\right)\right]=\overset{2}{\underset{j=0}{\sum }}\omega_{ij}\varphi \left(y_{i}-g_{i}\right)$$

where *ω_ij _*is the conditional probability of QTL genotype *j *given the marker genotype of progeny *i*.

Thus, the estimator of the parameters is

(4) $$\hat{\text{Θ}}=\arg\;\underset{\text{Θ}}{\min}\left(E_{e}\right)≅\arg\;\underset{\text{Θ}}{\min}\left(\frac{1}{2\sqrt{\pi \sigma^{2}}}-\frac{2}{N}\overset{N}{\underset{i=1}{\sum }}\overset{2}{\underset{j=0}{\sum }}\omega_{ij}\frac{1}{2\sqrt{\pi \sigma^{2}}}e^{{}^{-\frac{{\left(y_{i}-g_{j}\right)}^{2}}{2\sigma^{2}}}}\right)$$

In practice, the genomic location of a QTL is estimated by scanning positions across the genome. When the QTL is assumed to exist between the two markers, the *E_e _*is approximated twice, one by the LLN and the other by the calculation of *φ*(*e_i_*). However, if the QTL is scanned at a marker position, only the approximation by the LLN is needed because no mixture density is used in this situation. The energy function at the marker position *E_em _*is expressed as

(5) $$E_{em}=\frac{1}{2\sqrt{\pi \sigma^{2}}}-\frac{2}{N}\overset{N}{\underset{i=1}{\sum }}\varphi \left(e_{i}\right)=\frac{1}{2\sqrt{\pi \sigma^{2}}}-\frac{2}{N}\overset{2}{\underset{k=0}{\sum }}\overset{N_{k}}{\underset{i=1}{\sum }}\frac{1}{2\sqrt{\pi \sigma^{2}}}\;e^{-\frac{{\left(y_{i}-g_{k}\right)}^{2}}{2\sigma^{2}}}$$

where N_k _is the number of progeny in the marker genotype group *k *and $\underset{k}{\sum }N_{k}=N$.

### Phenotype data based L_2_E method (pL_2_E)

Unlike the error density, the phenotype data density contains a mixture of density functions each corresponding to a different QTL genotype. Also, because each marker genotype group *k *(*k *= 1,...,9 for two markers) has a different probability of linking with the QTL genotypes, the phenotype density is marker-dependent. The density for marker genotype *k *is expressed as

(6) $$\varphi_{k}\left(y_{i}\right)=\overset{2}{\underset{j=0}{\sum }}\omega_{kj}\varphi \left(y_{i}|g_{j},\sigma^{2}\right),$$

where *ω_ij _*is the conditional probability of QTL genotype *j *given the marker genotype *k*. From Eq. (2), the energy function for marker genotype *k *is

$$E_{d}^{k}=\int {\left(\phi \left(y\right)\right)}^{2}-\frac{2}{N_{k}}\overset{N_{k}}{\underset{i=1}{\sum }}\phi_{k}\left(y_{i}\right)=\int {\left(\overset{2}{\underset{j=1}{\sum }}\omega_{kj}\phi_{j}\right)}^{2}dy-\frac{2}{N_{k}}\overset{N_{k}}{\underset{i=1}{\sum }}\overset{2}{\underset{j=0}{\sum }}\omega_{kj}\frac{1}{2\sqrt{\pi \sigma^{2}}}e^{-\frac{{\left(y_{i}-g_{j}\right)}^{2}}{2\sigma^{2}}}$$

Notice that

$$\int {\left(\overset{2}{\underset{j=0}{\sum }}\omega_{kj}\varphi_{j}\right)}^{2}dy=\int {\left[\overset{2}{\underset{j=0}{\sum }}\omega_{kj}\frac{1}{\sqrt{2\pi \sigma^{2}}}e^{-\frac{{\left(y_{i}-g_{j}\right)}^{2}}{2\sigma^{2}}}\right]}^{2}dy=\frac{1}{\sqrt{2\pi \sigma^{2}}}\left[\overset{2}{\underset{j=0}{\sum }}\omega_{kj}^{2}+2\underset{i\neq j}{\sum }\omega_{ki}\omega_{kj}e^{-\frac{{\left(g_{i}-g_{j}\right)}^{2}}{4\sigma^{2}}}\right]$$

Thus, we have

$$E_{d}^{k}=\frac{1}{\sqrt{2\pi \sigma^{2}}}\left[\overset{2}{\underset{j=0}{\sum }}\omega_{kj}^{2}+2\underset{i\neq j}{\sum }\omega_{ki}\omega_{kj}e^{-\frac{{\left(g_{i}-g_{j}\right)}^{2}}{4\sigma^{2}}}-\frac{2\sqrt{2}}{N_{k}}\overset{N_{k}}{\underset{i=1}{\sum }}\overset{2}{\underset{j=0}{\sum }}\omega_{kj}\frac{1}{\sqrt{2\pi \sigma^{2}}}e^{-\frac{{\left(y_{i}-g_{j}\right)}^{2}}{2\sigma^{2}}}\right].$$

When a QTL is assumed to be at a marker position, *φ_k_*(*y_i_*) = *φ_k_*(*y_i _*| *m_j_*), which is not in a mixture form. The $E_{d}^{k}$ at a marker position can be simplified as:

$$E_{dm}^{k}=\frac{1}{\sqrt{2\pi \sigma^{2}}}-\frac{2}{N_{k}}\overset{2}{\underset{j=0}{\sum }}\overset{N_{kj}}{\underset{i=1}{\sum }}\frac{1}{\sqrt{2\pi \sigma^{2}}}e^{-\frac{{\left(y_{i}-g_{j}\right)}^{2}}{2\sigma^{2}}}$$

To combine the information from all nine marker genotypes, we take a weighted sum of the marker energy functions to calculate an overall energy function for phenotype data (*E_d_*) as

$$E_{d}=\overset{9}{\underset{k=1}{\sum }}h\left(N_{k}\right)E_{d}^{k}$$

or

$$E_{dm}=\overset{9}{\underset{k=1}{\sum }}h\left(N_{k}\right)E_{d}^{k}\;\text{for marker positions}.$$

Here *h(x) *is a monotone increasing function with respect to *x*. The reason for choosing such an *h *function is that the more progenies in one marker group, the better approximation accuracy achieved by the LLN, and the more weights should be put on this group. To determine the exact form of *h*, we need to use the formulation for eL_2_E. Because at marker positions, the eL_2_E and pL_2_E approaches use exactly the same information for the derivation of energy function, they should agree at those positions. Therefore, a comparison between the energy functions in (5) and (7) for eL_2_E and pL_2_E at marker positions suggests that *h(N_k_) = N_k_/N *where *N *is the total number of progeny. By using this form of *h(N_k_)*, the estimators for the unknown parameters in *E_d _*is

$$\begin{array}{c} \hat{\text{Θ}}=\arg\;\;\underset{\text{Θ}}{\min}\left(E_{d}\right)=\arg\;\;\underset{{}_{\text{Θ}}}{\min}\left[\overset{9}{\underset{k=1}{\sum }}N_{k}E_{d}^{k}/N\right]=\arg\;\;\underset{\text{Θ}}{\min}\left[\overset{9}{\underset{k=1}{\sum }}N_{k}E_{d}^{k}\right]\\ =\arg\;\underset{\text{Θ}}{\min}\left\{\frac{1}{\sqrt{\sigma^{2}}}\overset{9}{\underset{k=1}{\sum }}\left[\frac{N_{k}}{2\sqrt{2}}\left(\overset{2}{\underset{j=0}{\sum }}\omega_{kj}^{2}+2\underset{i\neq j}{\sum }\omega_{ki}\omega_{kj}e^{-\frac{{\left(g_{i}-g_{j}\right)}^{2}}{4\sigma^{2}}}\right)-\frac{1}{\sqrt{\bar{\sigma^{2}}}}\overset{N}{\underset{i=1}{\sum }}\overset{2}{\underset{j=0}{\sum }}\omega_{ij}e^{-\frac{{\left(y_{i}-g_{j}\right)}^{2}}{2\sigma^{2}}}\right]\right\} \end{array}$$

## Hypothesis testing

The existence of a significant QTL can be tested by the following hypotheses:

H_0_: g_0 _= g_1 _= g_2_H_1_: Not all equalities in H_0 _hold.

For these hypotheses, we can find their corresponding L_2_E estimates, $\Theta_{H_{0}}$ and $\Theta_{H_{1}}$, and energies, $E_{H_{0}}$ and $E_{H_{1}}$, respectively. Analogous to the likelihood ratio (LR) test statistics, we define an energy difference (ED) test statistics for our hypothesis testing:

$$ED=E_{H_{0}}-E_{H_{1}}$$

Because the mixture of density functions is a larger family than its composite density functions, the $E_{H_{1}}$ is minimized over a larger space than the $E_{H_{0}}$. Thus, $E_{H_{1}}$ should always be smaller than the $E_{H_{0}}$, i.e., the test statistics ED should always be positive. As typically done in genome-wide QTL mapping, a permutation test [27] is performed to determine the critical threshold value for ED.

## Results

### Monte Carlo simulation

We performed Monte Carlo simulation studies to examine the statistical properties of the L_2_E-based mapping model. Consider a sample size *N *from an F_2 _population, with which one chromosome segment was simulated with a length of 200 cM covered by 11 evenly spaced markers. Suppose there is a QTL responsible for a quantitative trait that is placed at 86 cM from the first marker on the left-hand side. Both the QTL and markers are assumed to be codominant. Three QTL genotypes are assumed to have different mean values, with a common variance (which is scaled according to a given heritability).

**Figure 2:**
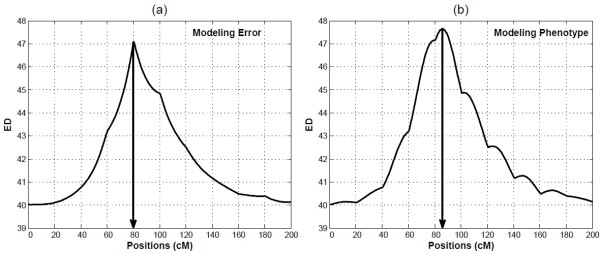
**Comparison between the two implementations of the L_2_E methods by simulation**. (a) Using the true density of the error term. (b) Using the true density of the observed data. The arrows to the x-axe indicate the peak of the ED profile. The true position of the QTL is at 86 cM from the left end of the simulated chromosome.

By scanning the simulated chromosome with a step size of 2 cM from the left end to the right end, the ED values were calculated and smoothed. Figure 2 shows two typical ED profiles obtained by modelling the error density (Figure 2A) or the phenotype density (Figure 2B). The peak value from modelling the error density always occurs at a marker position, although the true QTL location is placed between the fifth and sixth markers, whereas the method by using the phenotype density can find a peak ED value close to the true QTL location, suggesting that the pL_2_E approach performs better than the eL_2_E. Therefore, for the simulation studies and real-data analysis, the pL_2_E method will be used. This is reasonable because the derivation of eL_2_E involves two approximations but pL_2_E involves only one by the LLN. For the ease of notation, hereinafter L_2_E means pL_2_E.

Additional simulations were performed to examine the statistical properties of the L_2_E method, under different sample sizes (*N *= 100, 200, 400) and heritabilities (*H*^2 ^= 0.1, 0.2, 0.4). In each case, 100 replicates were run to evaluate the consistency and efficiency of the mapping methods. First, we consider simulation scenarios where error distributions in model (1) are normally distributed without any outlier data; *i.e*., the normal distribution is the true model. Because the simulation results from different sample sizes display similar patterns, here we only show the result for N = 400, which is tabulated in Table 1. Both L_2_E and traditional ML methods obtained consistent estimators and similar standard errors for the genetic effect parameters. However, the ML estimators have better efficiency as evidenced by smaller MSEs. This is expected because under the true model the MLE is asymptotically efficient.

**Table 1:** Simulation scenario 1.

		*H*^2 ^= 0.4		*H*^2 ^= 0.2		*H*^2 ^= 0.1	
Parameter	True Value	L_2_E	ML	L_2_E	ML	L_2_E	ML
*g*_2_	35	35(0.0685)	35(0.0514)	35.1(0.1061)	35(0.0833)	35.2(0.1653)	35.2(0.1396)
*g*_1_	30	30(0.0332)	30(0.0286)	30.1(0.0706)	30.1(0.0522)	30(0.1087)	30(0.0909)
*g*_0_	25	25(0.0724)	25.1(0.057)	25.1(0.0881)	25.1(0.0768)	24.9(0.1489)	24.8(0.1118)
sigma	4.3	4.3(0.0228)	4.3(0.0165)				
sigma	7.1			7.0(0.0344)	7.1(0.0282)		
sigma	10.6					10.4(0.0548)	10.6(0.0375)
Position	86	85.7(0.1386)	85.8(0.101)	85.9(0.2335)	86.4(0.1433)	86.0(0.5101)	85.8(0.2537)

The L_2 _and ML estimates of QTL parameters from an F2population of 400 individuals for the phenotypic data simulated from normal distributions. Numbers in the parentheses are the mean square errors (MSE) of the estimates

Second, we simulated scenarios where error distributions in model (1) are non-normal, using a *t*-distributions as error terms. In addition to different combinations of sample size and heritability, we also changed the degrees of freedom (df) of the *t*-distributed errors. When df is high (*e.g.*, df = 4), where the *t*-distribution approximate a normal distribution, the two methods perform similarly (Table 2). However, when df is low (*i.e.*, df = 2), where the *t*-distribution has much heavier tails than the normal distribution, the MLE method failed to give correct parameter estimates and yielded much larger standard errors. In the contrast, the L_2_E maintained the correct estimates with smaller standard errors. This demonstrates the robustness of the L_2_E method against model misspecification.

**Table 2:** Simulation scenario 2.

*t*-distribution:		df = 2		df = 3		df = 4	
Parameter	True Value	L_2_E	ML	L_2_E	ML	L_2_E	ML
*g*_2_	35	35.0(0.0168)	39.3(4.0988)	35.0(0.0139)	35.1(0.0185)	35.0(0.0117)	35.1(0.0133)
*g*_1_	30	30.0(0.0105)	30.0(0.0349)	30.0(0.0104)	30.0(0.0102)	30.0(0.0102)	30.0(0.0093)
*g*_0_	25	25.0(0.0163)	19.0(4.4676)	25.0(0.0158)	24.9(0.0192)	25.0(0.0131)	25.0(0.0133)
sigma	-	1.2(0.0083)	2.6(0.0971)	1.1(0.0077)	1.5(0.0337)	1.1(0.0056)	1.3(0.0099)
Position	86	86.4(0.0649)	85.6(0.0971)	86.1(0.053)	86.2(0.0591)	86.1(0.0609)	86.4(0.0498)

The L_2 _and ML estimates of QTL parameters from an F2population of 400 individuals with heritability of 0.4 for the phenotypic data simulated from t distributions. Numbers in the parentheses are the mean square errors (MSE) of the estimates

Third, we simulated experiments where data contains outlier data points. Because NP mapping is popular for traits with outliers [11], we compared the L_2_E model with both ML and NP approaches. The outliers were generated from another normal density on the upper tail of a mixture density. Different percentages of noise points (0, 5%, 10%, and 20%) were considered. The main results are shown in Table 3 with 10% outliers with noise mean at 45 and in Table 4 with 10% outliers with noise mean at 55. Our findings are summarized as follows: (1) With the existence of noise points, the L_2_E estimators are consistent but the ML estimators become biased towards the direction of the outliers. As the outliers move further away from the true density (from 45 to 55), the ML estimators perform significantly worse, but the L_2_E estimators stay consistent with very little impact. (2) As the heritability becomes smaller and smaller, the difference between the two methods becomes less. This is because the variation of the mixture density increases with decreasing heritability and, thus, the relative positions of the outliers become closer. This is consistent with the point (1) (3) L_2_E and NP methods show similar robustness to the outliers. However, the L_2_E method maintains the interpretability of a parametric model and gives accurate estimates of genetic effects. Overall, the simulation results demonstrate that the L_2_E method is preferred to the MLE and NP methods when the true model is misspecified or non-ignorable outliers exist.

**Table 3:** Simulation scenario 3.

		*H*^2 ^= 0.4			*H*^2 ^= 0.2			*H*^2 ^= 0.1		
**Parameter**	**True Value**	**L_2_E**	**ML**	**NP**	**L_2_E**	**ML**	**NP**	**L_2_E**	**ML**	**NP**
										
*g*_2_	35	35.3(0.0709)	35.9(0.0606)	-	35.7(0.1028)	35.9(0.0905)	-	36(0.1646)	36(0.1404)	-
*g*_1_	30	30.1(0.0335)	31.4(0.0389)	-	30.7(0.074)	31.5(0.0573)	-	31(0.1108)	31.4(0.0916)	-
*g*_0_	25	25(0.0696)	26.7(0.0774)	-	25.4(0.0911)	26.8(0.0881)	-	25.8(0.1628)	26.6(0.1244)	-
sigma	4.3	4.7(0.0238)	6.2(0.022)	-						-
sigma	7.1				7.6(0.0386)	8.3(0.0312)				
sigma	10.6							11.1(0.0567)	11.5(0.0376)	
Position	86	85.5(0.1466)	85.2(0.1712)	86.7(0.1387)	86(0.2272)	85.1(0.2528)	85.9(0.2562)	85.7(0.4935)	86.6(0.362)	85.4(0.3452)

The L_2 _and ML estimates of QTL parameters from an F_2 _population of 400 individuals for the phenotypic data simulated from normal distributions containing 10% noise points with mean *g *= 45. Numbers in the parentheses are the mean square errors (MSE) of the estimates

**Table 4:** Simulation scenario 4.

		*H*^2 ^= 0.4			*H*^2 ^= 0.2			*H*^2 ^= 0.1		
**Parameter**	**True Value**	**L_2_E**	**ML**	**NP**	**L_2_E**	**ML**	**NP**	**L_2_E**	**ML**	**NP**
										
*g*_2_	35	35(0.0664)	36.8(0.0789)	-	35.1(0.1061)	35(0.0833)	-	36.1(0.1731)	36.8(0.1494)	-
*g*_1_	30	30(0.0325)	32.3(0.0514)	-	30.1(0.0706)	30.1(0.0522)	-	30.8(0.1156)	32.5(0.1054)	-
*g*_0_	25	25(0.0699)	27.7(0.0872)	-	25.1(0.0881)	25.1(0.0768)	-	25.4(0.1531)	27.4(0.1412)	-
sigma	4.3	4.6(0.0231)	8.4(0.0253)	-						
sigma	7.1				7.0(0.0344)	7.1(0.0282)	-			
sigma	10.6							11.5(0.0588)	12.8(0.0421)	-
Position	86	85.6(0.1419)	84.8(0.2242)	86.7(0.1426)	85.9(0.2335)	86.4(0.1433)	86.6(0.1737)	85.5(0.5162)	85(0.6221)	85.8(0.4071)

The L_2 _and ML estimates of QTL parameters from an F_2 _population of 400 individuals for the phenotypic data simulated from normal distributions containing 10% noise points with mean g = 55. Numbers in the parentheses are the mean square errors (MSE) of the estimates

### A worked example

Vaughn et al. [20] constructed a linkage map with 96 microsatellite markers for 502 F_2 _mice (259 males and 243 females) derived from two inbreed strains, the Large (LG/J) and Small (SM/J). This map has a total map distance of 1780 cM (in 19 linkage groups) and an average interval length of 23 cM. The F_2 _progeny was measured for body mass at 10 weekly intervals starting at age 7 days. The raw weights were corrected for the effects of each covariant due to dam, litter size at birth, parity, and sex [20].

**Figure 3:**
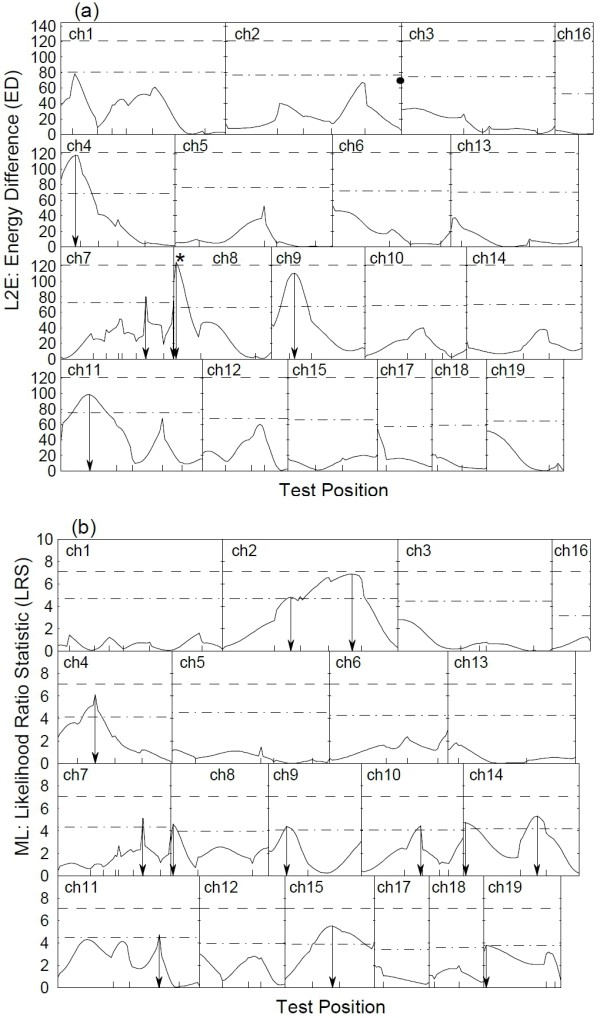
**L_2_E and MLE mapping of the mice data**. Genomic scanning profiles for mapping QTLs controlling the growth rate of body mass from weeks 5 to 10 by L_2_E (a) and ML approaches (b). The y-axes are the ED and LR test statistics, respectively. The dash dot line and the dash line are the chromosome-wide and genome-wide 0.05 cutoffs at the significant level of 0.05 based on the 1000 permutations, respectively. The x-axis ticks indicates the marker positions, the arrows to the × axes shows the genomic positions of the significant QTL at chromosome level, and the asterisk at chromosome 8 in the L_2_E profile marks a genome-wide significant QTL.

Our analysis here focuses on identifying QTLs that may affect the body mass growth rate from ages 5 weeks to 10 weeks, which is defined as body mass ratio between week 10 and week 5. On the right side of the empirical density of this trait (Figure 1), there is an obvious bump, suggesting the existence of some outliers. Both L_2_E and ML methods were applied to map this trait. The profiles of the two test statistics, energy difference (ED) and likelihood ratio statistic (LRS) across the whole mice genome is shown in Figure 3. The empirical distribution of test statistics were calculated on the basis of 1000 permutations and the 5% significance level was chosen.

Although the overall profiles of ED and LRS look similar, they did detect different significant QTLs. The ML method cannot identify any significant QTL at the genome level; however, the L_2_E method successfully detects one genome-wide significant QTL at 2 cM to the leftmost proximal marker on the chromosome 8. Coincidently, in 2005, Rance et al. [28] reported a significant QTL for the mature mice body mass located at 7 cM to the leftmost proximal marker on the chromosome 8, almost at the same location for the significant QTL identified here. Our finding hence further validates the existence of a significant QTL for mice body mass at the beginning of the chromosome 8. The genetic effects of the significant QTL identify by the L_2_E method are summarized in Table 5. This example shows the power of the L_2_E method to detect significant QTLs in practice.

**Table 5:** L_2_E mapping results of the mice data.

Chromosome	Map	Flanking Markers		QTL associated effects		
	position^a^	Marker 1	Marker 2	Additive^b^	Dominance^b^	%var^c^
8	2	D8Mit293	D8Mit25	0.012	-0.044	8.68

Significant QTL for body mass ratio between week 10 and week 5 in an F_2 _mouse population detected from the genome-wide interval mapping scan by the L_2_E and ML methods at the 0.05 significance level

^a^Map position = population-estimated position in cM from the leftmost proximal marker.

^b^Additive and dominance effects of the QTL

^c^%Var = percentage variance explained by the QTL.

## Discussion

Current mapping technologies allow us to dissect the variation of quantitative traits into individual genetic components (QTLs). Through this dissection the genetic architecture behind the quantitative traits can be elucidated, which provides a sound basis for future trait improvement. To better utilize the genomic data, considerable attention has been paid to develop powerful analytic methodologies that can increase the power, precision, and resolution of QTL mapping (8-16). Currently, almost all the QTL mapping methods proposed so far assume a parametric (mostly normal) distribution density of a trait. However, there is an increasing recognition of the limitation for the parametric assumption, given that in practice the true distribution of a trait is never known.

In this article, we propose a QTL mapping methodology based on the principle of L_2_E, which may allow the fitted model to be different from the true model. We derived two different implementation of the L_2_E method into the mapping framework and show how they are connected. The simulation studies suggest that the pL_2_E method works better than eL_2_E method and were used for our further analyses. Additional simulation studies were performed to test the statistical behaviour of the L_2_E-based mapping approach. The L_2_E method is more robust in the model choice at a cost of lower efficiency. For a "perfect" data, the ML performs better than the L_2_E. However, when the data contains noises, the L_2_E outperforms the ML. The relative efficiency of the L_2_E increases with increasing percentage of noises. In practice, it would be unrealistic for us to know the true model underlying the data, but it can be almost assured that no data is perfect. Thus, a better strategy is that the L_2_E method can first used to explore the data, with results compared with the MLE method.

This work is our first attempt to incorporate the principle of the integrated square errors into the genetic mapping framework. There are many areas that can be explored in the future, such as how to apply this principle to examine the gene-gene interaction or gene-environment interactions. The L_2_E method would be an excellent addition to the current toolbox of the QTL mapping.

## Conclusions

In this article, we derive a robust approach for genetic mapping of complex traits by incorporating the principal of the integrated square error into the general mapping framework. This approached, called the L_2_E mapping, automatically manipulates data points that are apparently outliers by giving them less weight in parameter estimation, and therefore yields more accurate estimation of QTL locations and effects. In the case where the data cleaning is not possible or very hard to do so, our new method could be a very beneficial choice. Simulation studies showed that in the presence of outliers, L_2_E method outperforms the traditional MLE and non-parametric methods in terms of both accuracy and efficacy of the parameter estimations. A real data analysis of the mice body mass data also demonstrates the usefulness and utilization of the new approach in a practical genetic setting. We strongly encourage researchers to explore both the L_2_E and MLE mapping procedures in practice.

## Authors' contributions

SW carried out the analysis, prepared and drafted the manuscript. GF participated in the design of the study. YC initiated the project design. ZW participated in the design of the study. RW initiated and established the overall project design, prepared and drafted the manuscript. All authors read and approved the final manuscript.
